# Coordinated regulation of IGF1R by HIF1α and HIF2α enhances chemoresistance in glioblastoma

**DOI:** 10.3389/fphar.2025.1575332

**Published:** 2025-04-11

**Authors:** Bin Liao, Pan Wang, Sheng Gong, Lu Zhao, Jie Liu, Nan Wu

**Affiliations:** ^1^ Chongqing Medical University, Chongqing, China; ^2^ Department of Neurosurgery, Chongqing Research Center for Glioma Precision Medicine, Chongqing General Hospital, Chongqing University, Chongqing, China

**Keywords:** glioblastoma multiforme, HIF1α, HIF2α, IGF1R, hypoxic microenvironment, chemoresistance, temozolomide

## Abstract

**Background:**

This study investigates whether Hypoxia-Inducible Factor 1 alpha (HIF1α) and Hypoxia-Inducible Factor 2 alpha (HIF2α) coordinately regulate insulin-like growth factor 1 receptor (IGF1R) expression, thereby influencing chemosensitivity in glioblastoma multiforme (GBM).

**Methods:**

We analyzed the expression and correlation of HIF1α, HIF2α, and IGF1R in glioma using The Cancer Genome Atlas (TCGA) and Chinese Glioma Genome Atlas (CGGA) databases. Immunohistochemistry (IHC) was performed to detect the expression of HIF1α, HIF2α, and IGF1R in GBM tissues and cells, as well as oxygen tension. Cell cycle analysis, apoptosis assays, lactate dehydrogenase (LDH) release measurements, Western blotting, and xenograft tumor models were employed to explore the synergistic regulation of IGF1R by HIF1α and HIF2α, focusing on activation of the PI3K/AKT signaling pathway and its contribution to GBM drug resistance. Chromatin immunoprecipitation-quantitative PCR (ChIP-qPCR) and dual-luciferase reporter assays were used to investigate the binding sites of HIF1α and HIF2α involved in regulating IGF1R.

**Results:**

Our study demonstrated that HIF1α and HIF2α were highly expressed in GBM tissues and hypoxia-cultured cells, and their expression positively correlated with IGF1R expression. Simultaneous knockout of HIF1α and HIF2α in GBM cells resulted in the highest LDH release and apoptosis rates under hypoxic conditions, accompanied by the most significant decrease in IGF1R, p-PDK1, and p-AKT levels. Knockout of IGF1R in tumor cells under hypoxia led to an increas of LDH release and apoptosis rates, and reduced phosphorylation of PDK1 and AKT. In addition, we demonstrated that HIF1α and HIF2α promoted IGF1R expression by binding to a specific hypoxia response element (HRE) sequence (5′-GAACGTGCCT-3′) within the IGF1R promoter using dual-luciferase reporter system.

**Conclusion:**

Glioblastoma cells, residing within a hypoxic microenvironment, exhibit high expression of HIF1α and HIF2α. These transcription factors promote the upregulation of IGF1R, which subsequently activates the PI3K/AKT signaling pathway. This activation, in turn, promotes cell proliferation and chemoresistance, ultimately contributing to tumor malignancy.

## 1 Introduction

Glioblastoma multiforme (GBM), the most malignant type of intracranial tumor, is associated with a poor prognosis (Ostrom et al., 2023). The current standard treatment for GBM involves surgical resection combined with radiotherapy and chemotherapy. However, despite these treatments, the prognosis remains dismal, with a median survival rate of only 14–16 months (Bou-Gharios et al., 2024; Valerius et al., 2024). This poor outcome is largely attributed to the hypoxic microenvironment of gliomas. The hypoxic tumor microenvironment lead to malignant phenotype, such as drug resistance, invasion, and proliferation (Wang et al., 2017b; Greenwald et al., 2024). Under hypoxic conditions, hypoxia-inducible factors (HIFs), particularly HIF1α and HIF2α, are stabilized and activated. These factors coordinately regulate the expression of multiple target genes, thereby activating the HIF signaling pathway (Wang et al., 2022; Sharma et al., 2023).

The insulin-like growth factor 1 receptor (IGF1R) is a transmembrane receptor tyrosine kinase that plays a crucial role in the development and progression of various cancers, including gliomas (Wang et al., 2023; Li et al., 2024). In GBM, the IGF1R-related signaling pathway has been shown to be closely associated with resistance to radiotherapy and chemotherapy, making it a key target for therapeutic intervention (Svalina et al., 2016; Liu et al., 2024). Studies have demonstrated that IGF1R activation promotes cell proliferation and therapy resistance, thereby enhancing the malignant phenotype of tumors (Yu et al., 2019; Wang et al., 2020). HIF1α and HIF2α, through complex mechanisms, synergistically increase IGF1R expression and activate downstream signaling pathways, such as the PI3K/AKT signaling pathway, which promotes tumor cell survival and proliferation, ultimately leading to resistance to temozolomide (TMZ) (Wen et al., 2023; Xiong et al., 2023). A deeper understanding of the mechanisms underlying the HIF1α/HIF2α-IGF1R axis in GBM progression is essential for developing novel therapeutic strategies.

## 2 Materials and methods

### 2.1 Public data collection

The expression of HIF1α and HIF2αin glioma and overall survival (OS) were analyzed using the Chinese Glioma Genome Atlas (CGGA) database (http://www.cgga.org.cn). The correlation between HIF1A and HIF2A with IGF1R in The Cancer Genome Atlas (TCGA) database (http://cancergenome.nih.gov/) was analyzed using the ggrepel, ggpubr, and ggplot2 packages in R.

### 2.2 Patients and specimens

GBM tissues (WHO IV) were obtained from surgical waste. Tumor grading was confirmed post-surgery by pathology, and ethical related to tumor tissues was approved by the ethics committee of Chongqing General Hospital (No. KY S2022-094-01).

### 2.3 Cell culture

HEK293T, U-87 MG, and U251 MG cells used in this study were purchased from the Cell Bank of the Chinese Academy of Sciences (www.cellbank.org.cn). Cells were cultured in DMEM/HG medium (Gibco, United States) supplemented with 10% fetal bovine serum (FBS, Gibco, United States). All cells were confirmed to be free of *mycoplasma* contamination to ensure the integrity of the experiments.

### 2.4 HIF and IGF1R knockout assays

sgRNAs targeting HIF1α, HIF2α, and IGF1R were used for plasmid construction (sgRNA oligonucleotide sequences are detailed in Supplementary Table S1). These were designed and synthesized using the CRISPR design program (http://crispr.mit.edu) and cloned into the lentiCRISPRv2 vector (Addgene, #52961, United States). The constructed plasmids were co-transfected with psPAX2 (Addgene #12260, United States) and pMD2. G (Addgene #12259, United States) into HEK293T cells to package lentivirus, which was then used to infect U87 and U251 cells for gene knockout. Western blotting was used to confirm the knockout of HIF1α, HIF2α, and IGF1R. To determine the effects of low HIF1α, HIF2α, and IGF1R expression on GBM cells, U87 and U251 cells were cultured under hypoxic conditions (1% O_2_) with TMZ, followed by detection of apoptosis and lactate dehydrogenase (LDH) release.

### 2.5 Cell treatments

Hypoxic (HYP) conditions used in our experiments consisted of 1% O_2_, 5% CO_2_, and 94% N_2_. For mRNA expression detection and hypoxia probe experiments, cells were cultured under HYP conditions for 12 h. For Western blotting, immunofluorescence, and cell cycle analysis, HYP group cells were cultured under HYP conditions for 72 h. For LDH release and flow cytometry apoptosis detection, HYP group cells were cultured under HYP conditions for 24 h, then stimulated with TMZ (400 or 800 μM) and continued to be cultured under HYP conditions for 72 h.

### 2.6 Immunohistochemistry detection

GBM tissues obtained from patients and tumor tissues from nude mice after intracranial tumor formation were used for IHC detection of HIF1α, HIF2α, IGF1R, and pimonidazole. Tissues were fixed in formalin, paraffin-embedded, and sectioned. Sections were deparaffinized in xylene, hydrated through a graded ethanol series, and finally immersed in distilled water. Antigen retrieval was performed by treating sections with citrate buffer at 95°C for 15 min. After washing with phosphate buffered saline (PBS), sections were incubated with primary antibodies overnight at 4°C (antibody information is provided in Supplementary Table S2). Sections were washed again with PBS, incubated with HRP-conjugated anti-mouse/rabbit secondary antibodies at room temperature for 2 h, and then incubated with 3,3′-diaminobenzidine (DAB) chromogen for approximately 1 min for color development. Finally, nuclei were counterstained with hematoxylin. Images were captured using an upright microscope (Olympus, United States).

### 2.7 Immunofluorescence detection

After culturing U87 and U251 cells under HYP conditions, the expression of HIF1α, HIF2α, and IGF1R was detected by immunofluorescence. Cells were fixed with 4% paraformaldehyde at 4°C for 30 min, washed three times with PBS for 5 min each, permeabilized with 0.5% Triton X-100 (Sigma, United States) at room temperature for 10 min, and then washed with PBS. Cells were blocked with goat serum (BOSTER, China) at room temperature for 2 h, incubated with primary antibodies overnight at 4°C (antibody information is provided in Supplementary Table S3), and then washed with PBS. Cells were incubated with Alexa Fluor 488-conjugated secondary antibodies (Beyotime, China) at room temperature in the dark for 2 h. After washing, cells were mounted with anti-fade mounting medium containing 4′,6-Diamidino-2′-phenylindole (DAPI) (Beyotime, China). Images were captured using an inverted fluorescence microscope (Olympus, United States).

### 2.8 Flow cytometry analysis

Cell apoptosis was detected using the FITC Annexin V Apoptosis Detection Kit I (BD Pharmingen, United States). Cells were digested with 0.25% trypsin, and 1 × 10^5^ cells were resuspended in 100 μL Annexin V binding buffer. Then, 5 μL Annexin V-FITC and 5 μL propidium iodide (PI) solution were added, mixed gently, and incubated at room temperature in the dark for 15 min. Apoptosis was detected using a flow cytometer (NovoCyte, United States). For cell cycle analysis, approximately 5 × 10^5^ cells were fixed in pre-chilled 75% ethanol at 4°C for 24 h, centrifuged at 1,000 rpm for 5 min, and the supernatant was discarded. Cells were resuspended in 500 μL PBS, and 5 μL PI and 5 μL ribonuclease (RNase) were added. Cells were incubated at 37°C for 30 min and stored at 4°C, with detection completed within 24 h.

### 2.9 Hypoxyprobe™-1 kit

The Hypoxyprobe™-1 kit (Hypoxyprobe, United States) was used to detect hypoxia in tissues or cells. Pimonidazole HCl was administered intraperitoneally (60 mg/kg), and nude mice were continued to be fed for 90 min. After perfusion with 4% paraformaldehyde, brains were removed, paraffin-embedded, sectioned, and stained using IHC. Pimonidazole HCl was added to the culture medium at a concentration of 100 μmol/mL, and cells were cultured under HYP conditions for 1 h, followed by staining using the immunofluorescence method.

### 2.10 Western blot

Total protein was extracted using radioimmunoprecipitation assay buffer (RIPA) (Beyotime, China), and protein concentration was determined using the Bicinchoninic Acid (BCA) Protein Assay Kit (Beyotime, China). Proteins were separated by sodium dodecyl sulfate - polyacrylamide gel electrophoresis (SDS-PAGE) and transferred to a membrane at 200 mA for 2 h. The membrane was blocked with 5% skim milk at room temperature for 2 h, incubated with primary antibodies overnight at 4°C (antibody details are provided in Supplementary Table S4), and then washed with tris buffered saline with tween^®^ 20 (TBST). The membrane was incubated with HRP-conjugated secondary antibodies (Beyotime, China) at room temperature for 2 h, washed again with TBST, and finally visualized using chemiluminescence.

### 2.11 Real-time quantitative polymerase chain reaction

Total RNA was extracted using the RNASimple Total RNA Kit (TIANGEN, China). Reverse transcription was performed using the MightyScript First Strand cDNA Synthesis Enzyme Mix (Sangon, China). Amplification was performed using 2X SG Fast qPCR Master Mix (Sangon, China) on a Bio-Rad CFX96 Real-Time PCR instrument (Bio-Rad, United States) with the following conditions: pre-denaturation at 95°C for 3 min; 40 cycles of 95°C for 3 s, 60°C for 20 s (annealing/extension/data acquisition). Primer sequences are listed in Supplementary Table S5.

### 2.12 Lactate dehydrogenase release

LDH release in culture was detected using the LDH Cytotoxicity Assay Kit (Beyotime, China). The supernatant was centrifuged at 400 × g for 5 min, and 120 μL was transferred to a new 96-well plate. Then, 60 μL LDH detection solution was added to each well, and the plate was incubated at room temperature in the dark for 30 min. Absorbance was measured at 490 nm using a Multiscan Spectrum (Thermo Scientific, United States). Cytotoxicity was calculated using the formula:

$$\text{Cytotoxicity }\left(\%\right)=\left(\text{Absorbance of treated sample}-\text{Absorbance of sample control}\right)\;/\left(\text{Absorbance of maximum enzyme activity}-\text{Absorbance of sample control}\right)\times 100.$$

### 2.13 Prediction of HIF1α and HIF2α binding sites in the IGF1R sequence

The binding sites of HIF1α and HIF2α in the IGF1R promoter sequence were predicted using the JASPAR database (http://jaspar.genereg.net/). The results indicated the presence of HIF1α and HIF2α binding motifs in the IGF1R promoter region.

### 2.14 Chromatin immunoprecipitation-qPCR

Chromatin immunoprecipitation was performed using the Pierce Agarose ChIP Kit (Thermo Scientific, United States). The experimental steps included cross-linking, cell pellet isolation, lysis, MNase digestion, immunoprecipitation, IP elution, and DNA recovery. Target gene fragments were obtained and used as templates for qPCR based on the predicted IGF1R promoter region (primer sequences are listed in Supplementary Table S6).

### 2.15 Detection of luciferase activity of the IGF1R promoter

The IGF1R promoter was cloned into the pGL3-basic luciferase reporter vector, and mutations were introduced at the HIF1α and HIF2α binding sites (completed by Shanghai Sangon Biotech). Normal or mutated IGF1R promoter reporter plasmids were co-transfected with the PRL vector (Promega, United States) into HEK293T-empty vector, HEK293T-HIF1α-KO, HEK293T-HIF2α-KO, and HEK293T-HIF1α/HIF2α-KO cells using Lipofectamine 2000. After 24 h, cells were lysed, and luciferase activity was detected using the Dual-Luciferase Reporter Assay Kit (Promega, United States). Promoter activity was measured by comparing luciferase levels.

### 2.16 *In vivo* study

30 BALB/c-Nude mice (male, 4–6 weeks old) of each group were used in this study, seven mice were employed to collect tumor tissues to detect protein expression, and other mice were used to analysis survival time. Empty vector cells, HIF1α-KO cells, HIF2α-KO cells, and HIF1α/HIF2α-KO cells (8 × 10^4^ cells) were inoculated into the brains of BALB/c-Nude mice. From day 3, TMZ (2 mg/kg) was administered intraperitoneally for three cycles (5 days of treatment followed by 2 days of rest). Tumor growth was monitored *in vivo* using the luciferase gene carried by the cells, and survival time was statistically analyzed.

All the animal procedures were approved by the ethics committee of ChongQing Medical University (No. IACUC-CQMU-2023-0167).

### 2.17 Statistical analysis

Statistical analysis was performed using GraphPad Prism 8 software. The significance between two groups was determined using Student’s t-test, while one-way ANOVA was used for multiple group comparisons. Survival analysis was performed using the Log-rank test, and Pearson correlation analysis was used to determine the correlation between HIF1α/HIF2α and IGF1R. A *P*-value < 0.05 was considered statistically significant.

## 3 Results

### 3.1 High expression of HIF1α and HIF2α in GBM tissues

**FIGURE 1:**
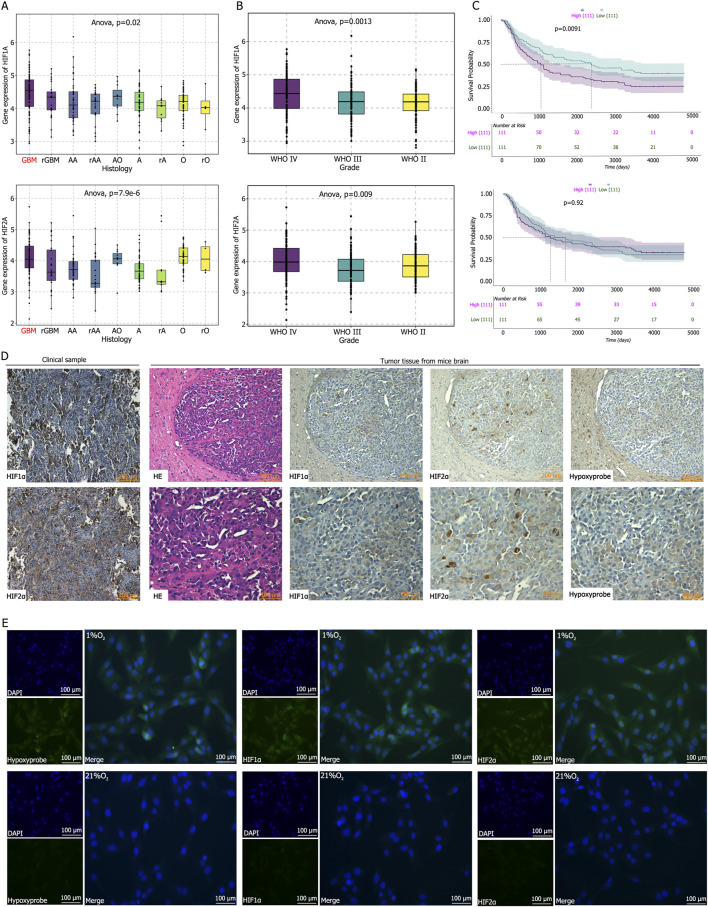
**(A–C)** Analysis of the CGGA database reveal that Hypoxia-Inducible Factor 1α (HIF1α) and Hypoxia-Inducible Factor 2α (HIF2α) are highly expressed in glioblastoma, with HIF1α expression positively correlated with glioma grade and negatively correlation with patient survival time. **(D)** Immunohistochemical staining demonstrated high expression levels of HIF1α and HIF2α in clinical GBM tissues and in tumor tissues from mice following intracranial tumor implantation. Hypoxia probe detection indicated low oxygen partial pressure within the intracranial tumor-bearing tissues of mice. **(E)** Immunofluorescence staining and hypoxia probe detection after culturing U87 cells in 21% O_2_ or 1% O_2_ for 72 h showed that the oxygen partial pressure in cells cultured in 1% O_2_ was significantly lower than that in the control group, and the expression levels of HIF1α and HIF2α were significantly higher than those in the control group.

The CGGA database indicates that HIF1α and HIF2α are highly expressed in gliomas (Figure 1A), with their expression levels gradually increasing with tumor grade, reaching the highest levels in glioblastoma (Figure 1B). Survival analysis reveals a negative correlation between HIF1α expression and survival, whereas HIF2α expression shows no statistically significant correlation with survival, although patients with low HIF2α expression tend to have prolonged survival (Figure 1C). Immunohistochemical staining of clinical GBM tissues (pathologically confirmed as WHO grade IV) demonstrated high expression of HIF1α and HIF2α (Figure 1D). Hypoxia probe detection in tumor-bearing mice reveald low oxygen partial pressure in tumor tissues, accompanied by high expression of HIF1α and HIF2α (Figure 1D). Immunofluorescence analysis of U87 cells cultured under normoxic or hypoxic conditions showed elevated hypoxia probe signals and high expression of HIF1α and HIF2α in hypoxic cultures, whereas normoxic conditions result in low expression of hypoxia probes, HIF1α, and HIF2α (Figure 1E).

### 3.2 HIF1α and HIF2α regulated GBM cell proliferation and apoptosis

**FIGURE 2:**
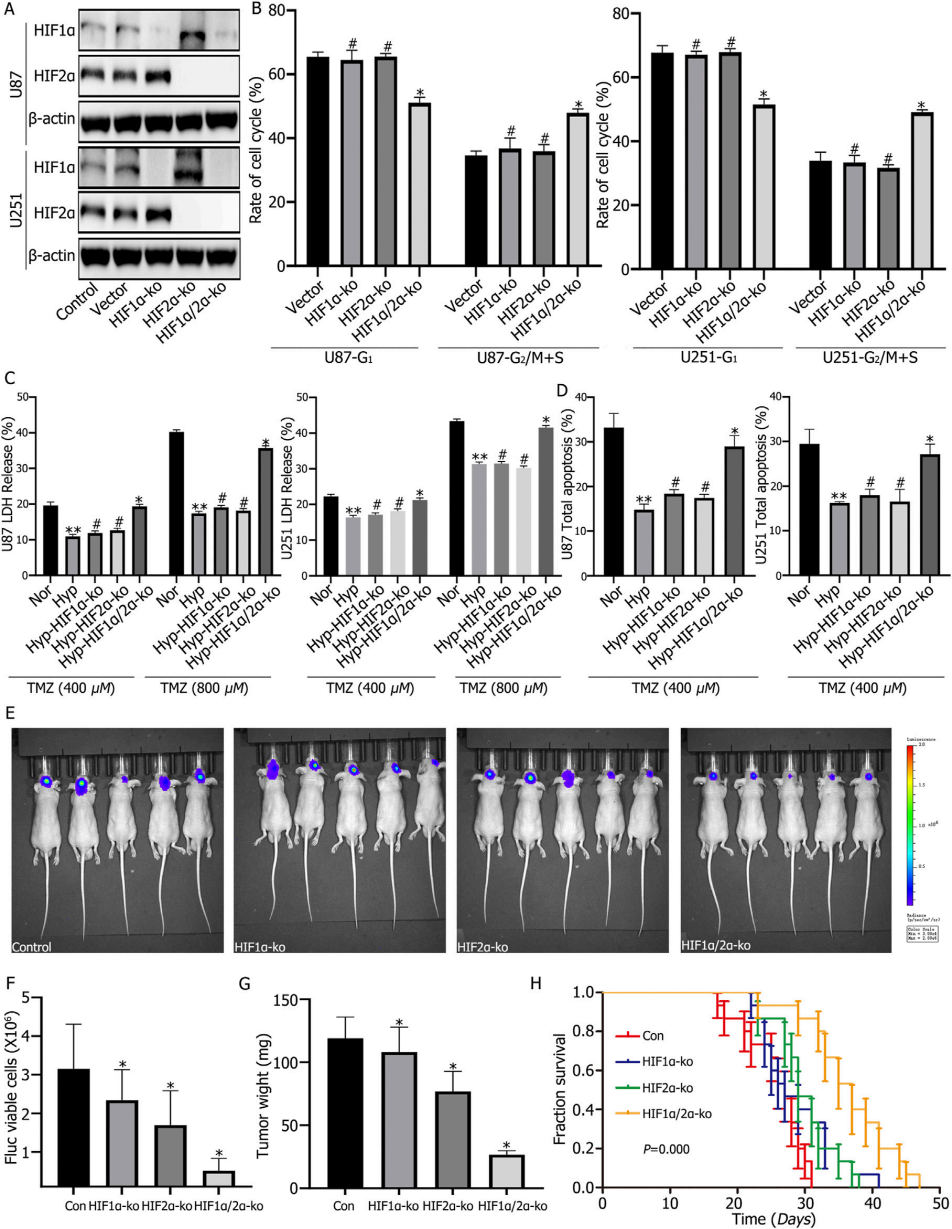
**(A)** Western blot analysis confirmed the successful knockout of HIF1α and HIF2α in U87 and U251 cells. It also demonstrated that HIF2α expression increased after HIF1α knockout, and vice versa, HIF1α expression increased after HIF2α knockout under hypoxic conditions. **(B)** Cell cycle analysis showed no significant changes in the cell cycle after single knockout of either HIF1α or HIF2α; however, simultaneous knockout significantly decreased the proportion of cells in the G_1_ phase and significantly increased the proportion in the G_2_/M + S phase. **(C, D)** Lactate Dehydrogenase (LDH) and apoptosis assays showed that both LDH release and apoptosis rates were reduced in the hypoxia group compared to the normoxia group. Single knockout of either HIF1α or HIF2α increased LDH release and apoptosis rates, while simultaneous knockout resulted in the highest LDH release and apoptosis rates. **(E–G)** Following intracranial orthotopic implantation of control, HIF1α-ko, HIF2α-ko, or HIF1α/HIF2α-ko cells (8 × 10^4^) and treatment with the same dose of Temozolomide (TMZ), tumor volume and weight were reduced in the HIF1α or HIF2α single knockout groups. The smallest tumor volume and weight were observed in the HIF1α/HIF2α double knockout group. **(H)** With the same dose of TMZ treatment, the survival time of mice in the HIF1α or HIF2α single knockout groups was prolonged compared to the control group, and the survival time of mice in the HIF1α and HIF2α double knockout group was the longest. *P < 0.05 represents the difference between HIF2α/HIF2α-ko and vector control under hypoxia, **P < 0.01 represents the difference between normoxia and hypoxia-treated groups, ^#^ *P* > 0.05 represents the difference between vector and single HIF1α or HIF2α knockout under hypoxia, and all comparisons were determined using one-way ANOVA.

Western blot (WB) analysis confirmed successful knockout of HIF1α and HIF2α in U87 and U251 cells, revealing that HIF2α expression increases following HIF1α knockout, while HIF1α expression increases following HIF2α knockout under hypoxic conditions (Figure 2A). Cell cycle analysis indicated that individual knockout of HIF1α or HIF2α did not significantly alter the cell cycle; however, simultaneous knockout leaded to a significant decrease in the G_1_ phase and an increase in the G_2_/M + S phase (Figure 2B). Further assessment of LDH release and apoptosis showed that both were significantly reduced in hypoxic cultures compared to normoxic cultures. In hypoxic cultures, LDH release and apoptosis rates increased with rising TMZ concentrations; individual knockout of HIF1α or HIF2α increased LDH release and apoptosis rates, while simultaneous knockout resulted in the highest levels of LDH release and apoptosis (Figures 2C, D). Intracranial implantation of control cells, cells with individual knockout of HIF1α or HIF2α, and cells with simultaneous knockout of HIF1α and HIF2α, followed by intraperitoneal injection and continuous TMZ treatment (2 mg/kg) for 3 weeks, revealed reduced tumor volume and weight, as well as prolonged median survival time in the individual knockout groups (Con vs. HIF1α-ko vs. HIF2α-ko = 27 days vs. 27 days vs. 29 days). The simultaneous knockout group exhibited the smallest tumor volume and weight, along with the longest medial survival time about 37 days (Figures 2E–H).

### 3.3 HIF1α and HIF2α regulated IGF1R expression in GBM cells

**FIGURE 3:**
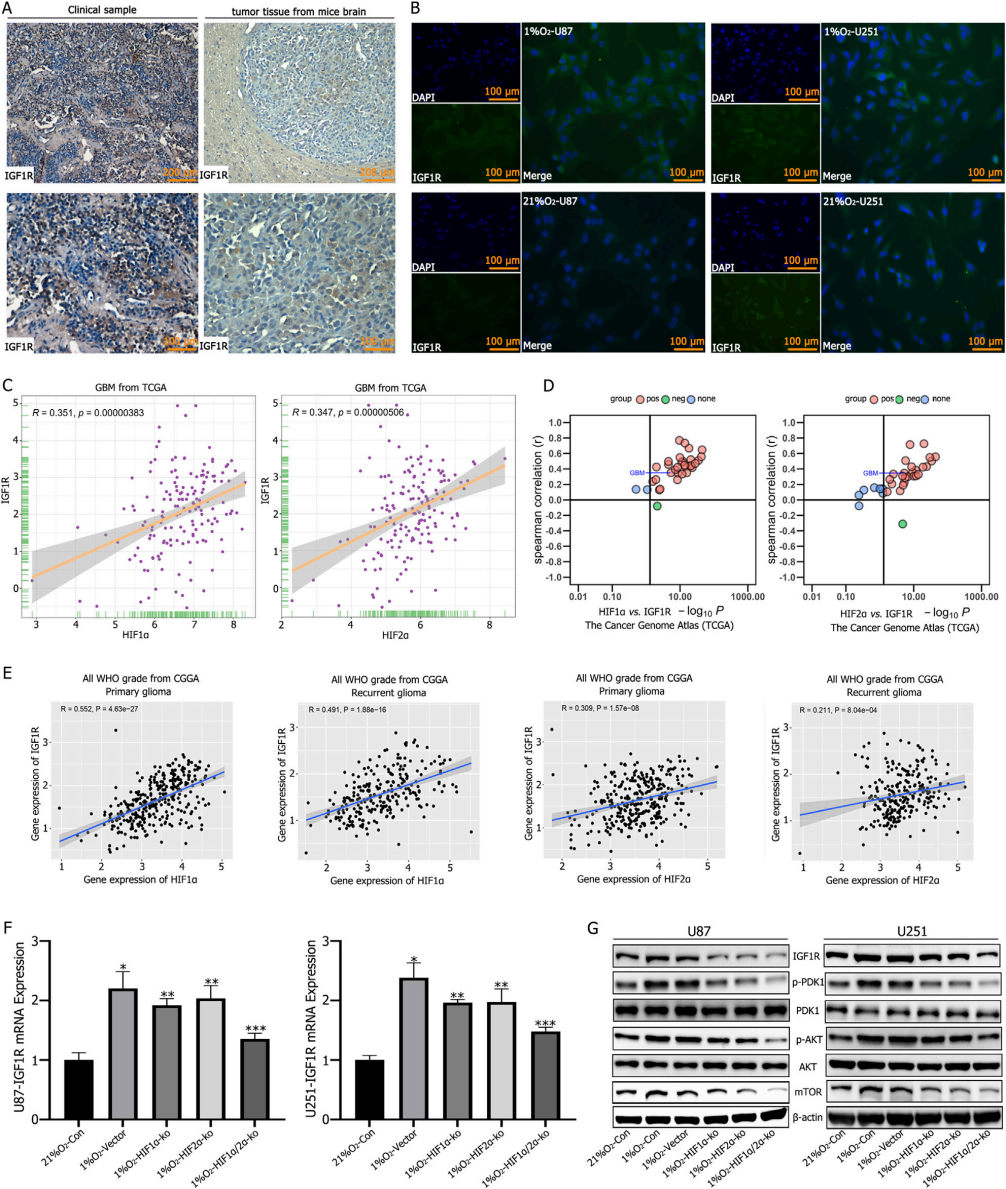
**(A)** Immunohistochemical staining showed high expression levels of Insulin-like Growth Factor 1 Receptor (IGF1R) in tumor tissues. **(B)** Immunofluorescence staining revealed low IGF1R expression in GBM cells under normoxic conditions, but significantly increased expression after 72 h of culture in a 1% O_2_ environment. **(C–E)** TCGA and CGGA database analyses showing a positive correlation between HIF1α,HIF2α,and IGF1R expression. **(F)** RT-qPCR analysis showed that IGF1R expression decreased after single knockout of either HIF1α or HIF2α; the most significant decrease in IGF1R expression was observed after simultaneous knockout of HIF1α and HIF2α. **(G)** Western blot analysis showed increased expression of IGF1R, p-PDK1, p-AKT, and mTOR after hypoxic culture. Single knockout of either HIF1α or HIF2α reduced the expression of IGF1R, p-PDK1, p-AKT, and mTOR, while simultaneous knockout of HIF1α and HIF2α resulted in the lowest expression levels of IGF1R, p-PDK1, p-AKT, and mTOR. *P < 0.05 represents the difference between normoxia and hypoxia-treated groups, **P < 0.05 represents the difference between vector and single HIF1α or HIF2α knockout under hypoxia, and ***P < 0.01 represents the difference between vector and both HIF1α and HIF2α knockout under hypoxia. All comparisons were determined using Student’s t-test.

Both clinical GBM specimens and tumor-bearing mouse models show high expression of IGF1R (Figure 3A). Immunofluorescence analysis of U87 and U251 cells cultured under normoxic or hypoxic conditions for 72 h revealed elevated IGF1R expression in hypoxic cultures, while normoxic cultures exhibited significantly reduced IGF1R expression (Figure 3B). The TCGA and CGGA databases indicate a positive correlation between HIF1α/HIF2α and IGF1R expression (Figures 3C–E). RT-qPCR analysis demonstrateed that individual knockout of HIF1α or HIF2α reduced IGF1R expression, while simultaneous knockout resulted in the most significant reduction in IGF1R expression (Figure 3F). Further validation by Western blotting showed that hypoxic culture reduced the expression levels of IGF1R, p-PDK1, p-AKT, and mTOR, with the most pronounced reduction observed following simultaneous knockout of HIF1α and HIF2α (Figure 3G).

### 3.4 IGF1R regulated GBM cell apoptosis via the PI3K-AKT pathway

**FIGURE 4:**
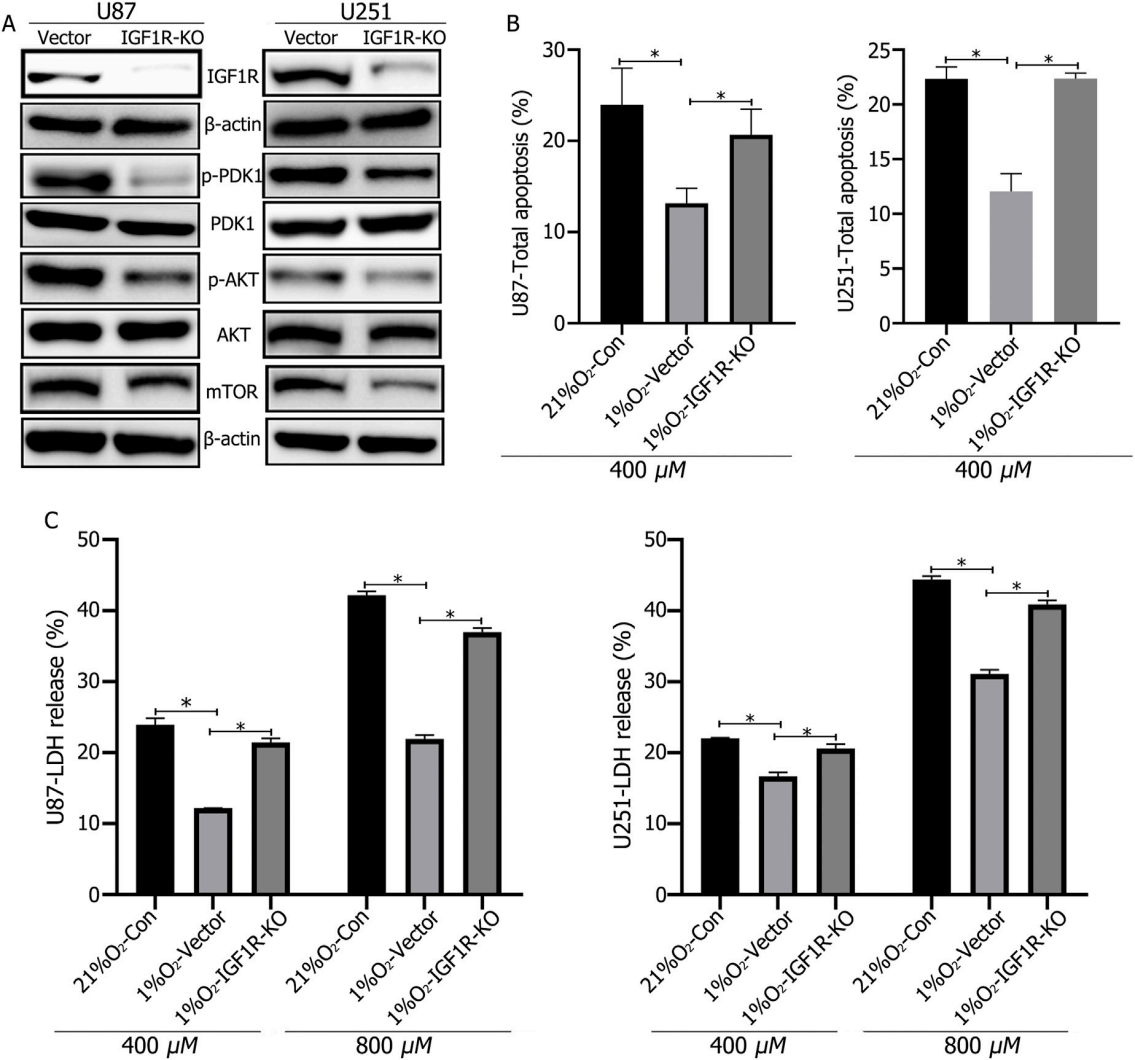
**(A)** Western blot analysis confirmed the successful knockout of IGF1R in U87 and U251 cells, and that IGF1R knockout led to decreased expression of p-PDK1, p-AKT, and mTOR. **(B, C)** LDH and apoptosis assays showed that IGF1R knockout increased cellular LDH release and apoptosis rates. *P < 0.05, determined using Student’s t-test.

Western blotting confirms successful IGF1R knockout. Hypoxic culture of IGF1R-knockout cells for 72 h results in significantly reduced expression of p-PDK1, and p-AKT, and mTOR, while PDK1 and AKT expression levels remain unchanged (Figure 4A). LDH release and apoptosis rates were significantly increased in IGF1R-knockout cells following TMZ treatment (Figures 4B, C).

### 3.5 Detection of HIF1α/HIF2α binding sites in IGF1R regulation

**FIGURE 5:**
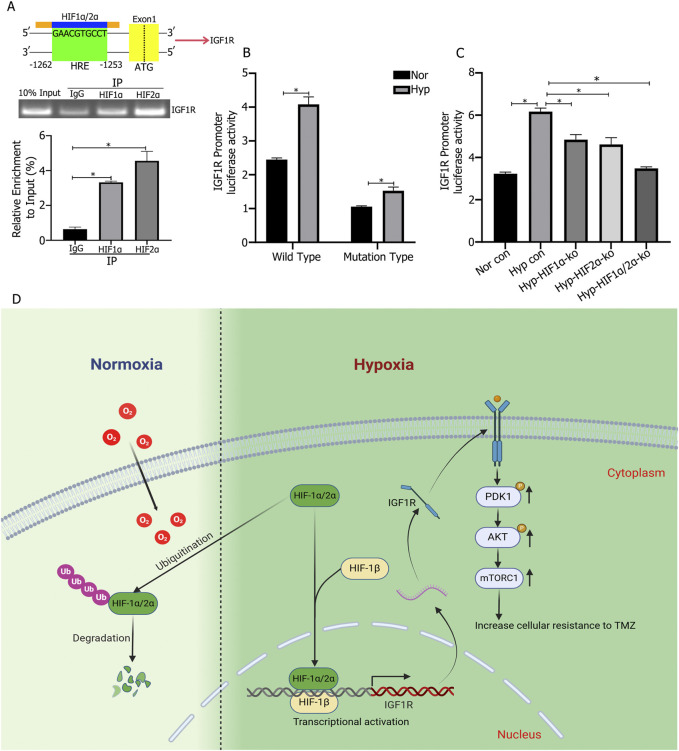
**(A)** Chromatin Immunoprecipitation-qPCR (ChIP-qPCR) experiments showed that HIF1α and HIF2α bind to the hypoxia response element (HRE) region (−1,253 to −1,262) of the IGF1R promoter and promote transcription. **(B)** IGF1R promoter activity was increased under hypoxic culture compared to normoxic culture, and the promoter activity significantly decreased after mutation of the binding site. **(C)** IGF1R promoter activity was lower in cells with single knockout of either HIF1α or HIF2α compared to control cells, and the lowest promoter activity was observed in cells with simultaneous knockout of HIF1α and HIF2α. **(D)** Under hypoxic conditions (1% O_2_), HIF1α and HIF2α promote high expression of IGF1R in cells. The highly expressed IGF1R activates the PI3K/AKT signaling pathway, thereby promoting cell proliferation and chemoresistance. *P < 0.05, determined using one-way ANOVA or Student’s t-test.

Analysis of the JASPAR database identified the core conserved sequence of the hypoxia response element (HRE) in the IGF1R promoter as 5′-GAACGTGCCT-3′ (−1,262 to −1,253), with a relative score of 0.957797 (Figure 5A). ChIP-qPCR experiments confirmed that HIF1α and HIF2α targeted the HRE region of the IGF1R promoter (Figure 5A). To validate the regulatory process, the wild-type IGF1R promoter activity detection plasmid (pGL3/basic-IGF1R-WT) and a mutant plasmid (pGL3/basic-IGF1R-MT) were constructed and transfected into HIF1α or HIF2α knockout cells to assess changes in luciferase activity. Under 1% O_2_ culture conditions, the MT plasmid transfection group exhibited significantly lower IGF1R promoter activity compared to the WT plasmid transfection group. In the WT plasmid transfection group, hypoxic culture significantly increased IGF1R promoter activity compared to normoxic culture, while no such difference was observed in the MT plasmid transfection group (Figure 5B). Additionally, in WT plasmid-transfected cells with individual knockout of HIF1α or HIF2α, IGF1R promoter activity decreased compared to control cells, while simultaneous knockout of HIF1α and HIF2α resulted in the lowest promoter activity (Figure 5C).

## 4 Discussion

Gliomas, a group of tumors originating from glial cells, are classified into different types and grades based on histological features and malignancy. Among these, GBM is the most common and malignant, classified as WHO grade IV (Louis et al., 2021; Weller et al., 2024). GBM is characterized by high heterogeneity, including genetic and molecular variations, and a complex tumor microenvironment that contributes to its high resistance to TMZ. Hypoxia in the tumor microenvironment is one of the key factors driving this resistance (Wang et al., 2017b; Valerius et al., 2024). Hypoxia, or low oxygen conditions, is a common feature of solid tumors, including gliomas, and we have confirmed through hypoxia probes that glioma tissues exist in a hypoxic environment. Previous studies have shown that hypoxia promotes tumor cell proliferation, inhibits apoptosis, and enhances stemness. *In vivo* experiments have also demonstrated that hypoxia-fed mice exhibit larger intracranial tumors and shorter survival (Wang et al., 2021).

At the molecular level, HIFs, particularly HIF1α and HIF2α, are key transcription factors that regulate cellular responses to low oxygen conditions. Under normoxic conditions, the HIF-α subunit is hydroxylated by prolyl hydroxylase (PHD), followed by ubiquitination and degradation. However, under hypoxia, PHD activity is inhibited, allowing the HIF-α subunit to stabilize and form a functional HIF transcription factor with the HIF-β subunit. The activated HIF enters the nucleus and binds to hypoxia response elements (HREs) in the promoter regions of target genes, thereby regulating gene expression and activating the HIF signaling pathway. In highly invasive brain tumors like GBM, these factors play a critical role in cell cycle regulation, proliferation, and therapy resistance. A study demonstrated that HIF1α is highly expressed in GBM cells, further promoting epithelial-mesenchymal transition (EMT), tumor invasion, and chemotherapy resistance, thereby exacerbating tumor malignancy (Liu et al., 2020). Regarding HIF2α, a study suggested that HIF2α primarily acts on glioma stem cells, promoting stemness, sphere-forming ability, and inhibiting apoptosis, thereby worsening tumor malignancy, a finding corroborated by *in vivo* experiments (Li et al., 2009). These observations have been further supported by subsequent studies (Grassi et al., 2020; Kumar et al., 2023). However, the synergistic regulation of glioma malignancy by HIF1α and HIF2α has been less explored.

Our research revealed that HIF2α expression increased in HIF1α-knockout cells under hypoxic conditions, while HIF1α expression increased in HIF2α-knockout cells. Cell cycle analysis showed that knocking out either HIF1α or HIF2α alone did not affect the cell cycle, but simultaneous knockout resulted in cells predominantly in the G_2_/M + S phase. Further TMZ intervention led to increased LDH release and apoptosis rates in cells with either HIF1α or HIF2α knockout, with the highest levels observed in cells with both HIF1α and HIF2α knockout. However, a limitation is that there are lack of statistical significance for HIF2α in survival analysis according to CGGA database, and this may be due to not enough patients data and grouping defect maybe another reason, and we will verify it in the future.

IGF1R, as a receptor for many exogenous factors, activates a series of signaling pathways upon binding to its ligands, promoting invasion, proliferation, and inhibiting apoptosis, thereby exacerbating tumor malignancy (Wang et al., 2023). For example, NCK1-AS1 overexpression increases IGF1R expression, further promoting glioma cell proliferation and resistance to radiotherapy and chemotherapy (Wang et al., 2020). Other studies have also indicated that IGF1R, regulated by certain miRNAs, promotes proliferation and invasion (Wang et al., 2017a). Our study found that IGF1R expression significantly increases under hypoxic conditions. However, the regulation of IGF1R by HIF1α and HIF2α has been less studied. A previous study from breast cancer showed HIF1α upregulated IGF1R expression, and promoted cancer cells proliferation, invasion and chemotherapy-resistance (Mancini et al., 2014). In addition, HIF1α is regulated synergistically with IGF1R in lung cancer through glycolysis and glutamine metabolism, result of including cancer malignant (Fujiki et al., 2017). However, few studies have studies the relationship between HIF2α and IGF1R. Accordingly, we conducted research and found that TCGA and CGGA databases indicate a positive correlation between HIF1α, HIF2α, and IGF1R expression in glioma tissues. Knockout of either HIF1α or HIF2α alone reduced IGF1R expression, with the most significant reduction observed when both HIF1α and HIF2α were knocked out, a result confirmed by Western blot analysis. Additionally, we identified the binding site of HIF1α and HIF2α on IGF1R promoter sequence as GAACGTGCCT. Mutating this binding site significantly reduced IGF1R promoter activity under hypoxic conditions. In summary, under hypoxia, HIF1α and HIF2α regulate downstream IGF1R expression in glioma cells.

The signaling pathways through which IGF1R, activated by HIF1α and HIF2α, promotes tumor cell proliferation and therapy resistance in glioma cells are also worth exploring. Previous studies have shown that increased IGF1R expression activates pathways such as PI3K/AKT (Quail et al., 2016; Nemati et al., 2022), MAPK (Benito-Jardón et al., 2019), and Wnt (Zhang et al., 2015), thereby promoting proliferation and inhibiting apoptosis, exacerbating tumor malignancy. Our research revealed that knocking out IGF1R in tumor cells and intervening with TMZ significantly increased LDH release and apoptosis rates. Western blot analysis showed decreased phosphorylation of PI3K/AKT signaling proteins p-PDK1, p-AKT, and mTOR, while PDK1 and AKT expression remained unchanged. Therefore, we conclude that under hypoxia, glioblastoma cells highly express HIF1α and HIF2α, which synergistically promote IGF1R expression, leading to the activation of downstream PI3K/AKT signaling through phosphorylation, thereby promoting cell proliferation and inhibiting apoptosis, exacerbating tumor malignancy (Figure 5D).

Therefore, combined HIF and IGF1R target therapy with TMZ is vital important to improve GBM patients prognosis. HIF inhibitors inhibit tumor growth and angiogenesis by interfering with the ability of tumor cells to adapt to hypoxic environments. Further studies confirmed that HIF inhibitor combined with TMZ can promote tumor cell apoptosis, synergistic with TMZ DNA damage, and promote tumor cell death (Schöning et al., 2017). For example, PT2385, as a selective inhibitor of HIF-2α, inhibits the transcriptional activity of HIF-2α by directly binding with HIF-2α protein and blocking its binding with HIF-1β (Arnaiz et al., 2021), but PT2385 is mainly used in the treatment of HIF-2α-related renal cell carcinoma (Courtney et al., 2018), and the therapeutic effect in other tumors is less studied. IGF1R inhibitors inhibit tumor cell proliferation and promote apoptosis by blocking related signaling pathways, thus increasing tumor chemotherapy sensitivity (Flanigan et al., 2013), such as OSI-906. As a selective IGF1R inhibitor, OSI-906 inhibits the growth and survival of tumor cells by blocking the tyrosine kinase activity of IGF1R and inhibiting the activation of downstream signaling pathways (Liu et al., 2022). Preclinical studies have shown that OSI-906 has antitumor activity in a variety of tumor models, especially in tumors with IGF1R overexpression (Jones et al., 2015), but it has been less studied in GBM. Therefore, based on our results, we will further explore the possibility of improving the prognosis of patients by combining targeted HIF-1α, HIF-2α and IGF1R proteins under the intervention of TMZ, so as to provide new targets and strategies for GBM treatment.

## 5 Conclusion

Glioblastoma exists in a hypoxic environment and highly expresses HIF1α and HIF2α, which bind to GAACGTGCCT to promote high expression of the IGF1R receptor. High IGF1R expression enhances the phosphorylation of PDK1 and AKT, activating the PI3K/AKT signaling pathway, thereby promoting cell proliferation and chemotherapy resistance, exacerbating tumor malignancy. Targeting the HIF-IGF1R axis represents a novel therapeutic approach to overcome glioblastoma proliferation and TMZ resistance. Developing effective inhibitors that simultaneously target HIF and IGF1R, along with implementing combination therapies, may significantly improve the prognosis of patients with this aggressive cancer, providing new strategies and targets for comprehensive GBM treatment.

## Data Availability

The raw data supporting the conclusions of this article will be made available by the authors, without undue reservation.
