# Supplementary material for: Coordinated regulation of IGF1R by HIF1α and HIF2α enhances chemoresistance in glioblastoma
Source: Front Pharmacol. 2025 Apr 11;16:1575332. doi: 10.3389/fphar.2025.1575332 (PMC12021886; doi:10.3389/fphar.2025.1575332)
Supplement: Supplementary file 1 [file Table1.docx]

Table S1 The sequences of sgRNA for knockout of HIF1α、HIF2α and IGF1R

| Target | Oligonucleotide sequence(5'-3') |
| --- | --- |
| HIF1α | GAACTCACATTATGTGGAAG |
| HIF2α | CTTGGAGGGTTTCATTGCCG |
| IGF1R | GATGATGCGATTCTTCGACG |
