# Supplementary material for: Coordinated regulation of IGF1R by HIF1α and HIF2α enhances chemoresistance in glioblastoma
Source: Front Pharmacol. 2025 Apr 11;16:1575332. doi: 10.3389/fphar.2025.1575332 (PMC12021886; doi:10.3389/fphar.2025.1575332)
Supplement: Supplementary file 2 [file Table2.docx]

Table S2 Primary antibodies used in Immunohistochemistry

| Antigens | Manufacturer | Catalogue numbers | Application |
| --- | --- | --- | --- |
| HIF1α | abcam | ab179483 | 1:500 |
| HIF2α | NOVUS | NB100-132 | 1:200 |
| IGF1R | Proteintech | 20254-1-AP | 1:100 |
| anti-pimonidazole antibody | Hypoxyprobe | PAb2627AP | 1:100 |
