# Supplementary material for: Coordinated regulation of IGF1R by HIF1α and HIF2α enhances chemoresistance in glioblastoma
Source: Front Pharmacol. 2025 Apr 11;16:1575332. doi: 10.3389/fphar.2025.1575332 (PMC12021886; doi:10.3389/fphar.2025.1575332)
Supplement: Supplementary file 3 [file Table5.docx]

Table S5 The sequences of primers used for qRT-PCR detection

| IGF1R | Forward(5'-3') | TGCTGACCTCTGTTACCTCTCCAC |
| --- | --- | --- |
|  | Reverse(5'-3') | \| GTCTTCTCACACATCGGCTTCTCC \| \| --- \| |
| β-Actin | Forward(5'-3') | ACCCGCCGCCAGCTCACC |
|  | Reverse(5'-3') | GGGGGGCACGAAGGCTCATC |
