# Supplementary material for: Coordinated regulation of IGF1R by HIF1α and HIF2α enhances chemoresistance in glioblastoma
Source: Front Pharmacol. 2025 Apr 11;16:1575332. doi: 10.3389/fphar.2025.1575332 (PMC12021886; doi:10.3389/fphar.2025.1575332)
Supplement: Supplementary file 5 [file Table4.docx]

Table S4 Primary antibodies used in western blotting

| Antigens | Manufacturer | Catalogue numbers | Application |
| --- | --- | --- | --- |
| HIF1α | abcam | ab179483 | 1:1000 |
| HIF2α | abcam | ab207607 | 1:1000 |
| IGF1R | Proteintech | 20254-1-AP | 1:1000 |
| AKT | CST | 2920S | 1:1000 |
| p-AKT | CST | 4060S | 1:1000 |
| PDK1 | CST | 5662S | 1:1000 |
| P-PDK1 | CST | 3438S | 1:1000 |
| mTOR | CST | 2983S | 1:1000 |
| β-actin | Sangon Biotech | [D191047-0100](https://store.sangon.com/productDetail?productInfo.code=D191047-0100) | 1:5000 |
